# Correction: Mofed et al. Construction of a Macrophage-Tropic Subtype C HIV-1-mGreenLantern Reporter Virus for Studies on HIV-1 Replication and the Impact of Methamphetamine. *Viruses* 2024, *16*, 1859

**DOI:** 10.3390/v18050568

**Published:** 2026-05-18

**Authors:** Dina Mofed, Angelo Mandarino, Xuhong Wu, Yuekun Lang, Anjali Gowripalan, Ganjam V. Kalpana, Vinayaka R. Prasad

**Affiliations:** 1 Department of Microbiology and Immunology, Albert Einstein College of Medicine, Bronx, NY 10461, USA; dina.mahmoud@einsteinmed.edu (D.M.); angelo.mandarino@gmail.com (A.M.); ylcgm@missouri.edu (Y.L.); anjali.gowripalan@gmail.com (A.G.); 2 Department of Genetics, Albert Einstein College of Medicine, Bronx, NY 10461, USA; xuhong.wu@einsteinmed.edu (X.W.); ganjam.kalpana@einsteinmed.edu (G.V.K.)

**Error in Figure**

In the original publication [1], Figure 4 contained an error introduced during the production process. The corrected version of Figure 4 is provided below. Specifically, there was image duplication in Figure 4B (column, 9 dpi, rows ISO and CCL2, and column, 30 dpi, rows ISO and CCL2). The authors state that the scientific conclusions are unaffected. This correction was approved by the Academic Editor. The original publication has also been updated.

## Figures and Tables

**Figure 4:**
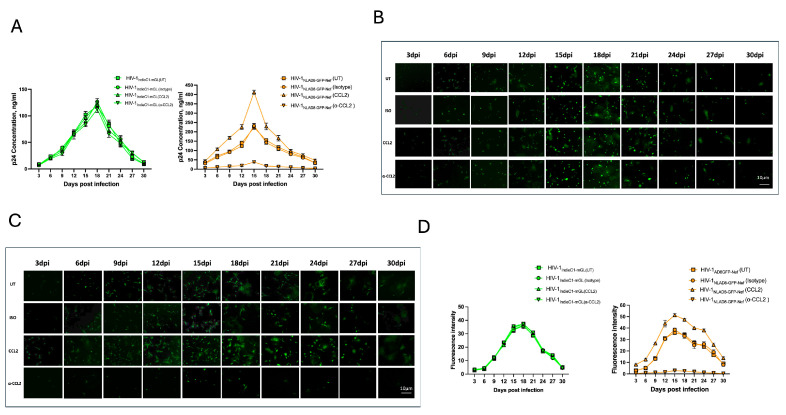
Effect of CCL2 on HIV-1 subtype C and B replication in MDMs. (**A**) MDMs were infected with HIV-1_IndieC1-mGL_ reporter virus (**Left**) or HIV-1_NLAD8-GFP-Nef_ (**Right**) and treated with CCL2, anti-CCL2, or isotype control antibody (see Methods). UT = no treatment. (**B**) Tracking the effect of CCL2 or anti-CCL2 on HIV-1_IndieC1-mGL_ reporter replication via fluorescence intensity. (**C**) Tracking the effect of CCL2 or anti-CCL2 on HIV-1_NLAD8-GFP-Nef_ replication in MDMs using fluorescence intensity. dpi, days post infection. Scale bar for (**B**,**C**) is as in the panel anti-CCL2, 30 dpi. (**D**) Measurement of fluorescence intensity in panels (**B**,**C**) for subtype C or subtype B reporter viruses with various treatments. Data are a mean of three experiments ± SEM (*n* = 3).
